# A practical route to β^2,3^-amino acids with alkyl side chains

**DOI:** 10.1186/s40064-015-1351-6

**Published:** 2015-09-25

**Authors:** Luigi Longobardo, Marina DellaGreca, Ivan de Paola

**Affiliations:** Dipartimento di Scienze Chimiche, Università degli Studi di Napoli Federico II, Via Cinthia 4, 80126 Naples, Italy; Dipartimento delle Scienze Biologiche, Università degli Studi di Napoli Federico II, Via Mezzocannone 16, 80134 Naples, Italy

**Keywords:** Beta-amino acids, Beta-amino nitriles, Alkylation, Homologation, Carbanions

## Abstract

Enantiopure *N*(Boc)-β^3^-amino nitriles, valuable synthetic intermediates in the multistep homologation of α-amino acids, were alkylated using *n*-BuLi as base. Alkylations afforded easily separable, almost equimolecular mixtures of diastereomeric *N*(Boc)-protected *syn* and *anti* β^2,3^-amino nitriles. Suitable manipulations of both cyano and amino groups eventually led to enantiopure *N*- and/or *C*-protected β^2,3^-amino acids. For example, methanolysis using conc. HCl gas in MeOH, provides *C*-protected β^2,3^ amino acids in excellent yields. This methodology is applied to the synthesis of a series *N*(Boc)-β^2,3^-dialkyl amino nitriles derived from l-phenylalanine, d-phenylalanine, l-valine and one *C*-protected β^2,3^ amino acid. We demonstrate an efficient procedure for the preparation of *anti* and *syn* β^2,3^-amino acids with alkyl side chains, from α-amino acids in reasonable yields.

## Background

Beta amino acids have shown great potential for a wide range of applications in many fields of organic chemistry in recent years (Juaristi and Soloshonok 2005). The growing interest in this class of compounds, which are widely used in medicinal chemistry forming new secondary structures and as valuable synthetic building blocks, can be better appreciated by database searching (e.g. Sci-Finder). Almost all β^3^-amino acids with proteinogenic side chains are now commercially available, but are quite expensive. Several synthetic procedures have been reported for the preparation of β-amino acids, and the field has been extensively reviewed (Cole 1994; Liu and Sibi 2002; Weiner et al. 2010).

**Fig. 1:**
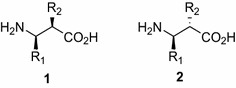
*Anti* (**1**) and *syn* (**2**) β^2,3^-amino acids

β^2,3^-Amino acids have two substituent at the α (C2) and β (C3) positions (Fig. 1). They are relatively rare in nature, although occur as substructures in several bioactive compounds and important metabolites (Juaristi and Soloshonok 2005). These β-amino acids bearing two side chains are of particular interest for the synthesis of β-peptides (oligomers of β-amino acids) in view of their conformation-inducing ability and thereby the ability to afford new foldamers (Seebach et al. 1999; Cheng et al. 2001; Martinek and Fülöp 2012).

β^2,3^-Amino acids may be either homochiral (anti- or *like*-β^2,3^-amino acids) or heterochiral (syn- or *unlike*-β^2,3^-amino acids) and it is noteworthy that, when included as building blocks in peptides, the former (**1**) afford predominantly helical structures, with all substituents in lateral positions, whereas their *syn* diastereomers (**2**) adopt an extended conformation, with formation of pleated sheets (Seebach et al. 1999; Balamurugan and Muraleedharan 2015).

A number of synthetic procedure have been proposed for the preparation of β^2,3^-amino acids and many of them have been reported recently in an excellent review (Kiss et al. 2015), but very few have been originate from amino acid precursors (Burgess et al. 1993) which have the obvious advantage of transferring pre-existing structural information, such as the nature of side chain and the chirality, into the final products. Among these, the Arndt-Eistert homologation is the most commonly used procedure (Podlech and Seebach 1995). The process involves the conversion of *N*-protected α-amino acid mixed anhydrides into the corresponding α-diazoketones using diazomethane, followed by the Wolff rearrangement. It has seen a resurgence in popularity in recent years due to the work carried out at ETH in Zurich, which has also identified new secondary structures by inserting several β^3^ residues in homologous peptide sequences (Seebach et al. 2004). However, this procedure is aimed at obtaining β^3^-amino acids and only involves intermediate α-diazoketones.

These intermediates have also been used in the preparation of α-methyl β^3^-residues via KHMDS/HMPA methylation, in modest yields (Yang et al. 2000). *Anti*-β^2,3^-amino acids can be prepared by the alkylation of β^3^-amino esters (Estermann and Seebach 1988; Cardillo et al. 1996; Capone et al. 2007). An alternative approach to the asymmetric synthesis of *syn* and *anti* β^2,3^-amino acids from non-amino acid precursors, exploits the conjugate addition of a chiral *N*-benzyl-*N*-(α-methylbenzyl) lithium amide to a specially prepared α,β-unsaturated ester, already bearing the side chains needed in the final product (Davies et al. 1994; Langenhan and Gellman 2003].

**Scheme 1:**
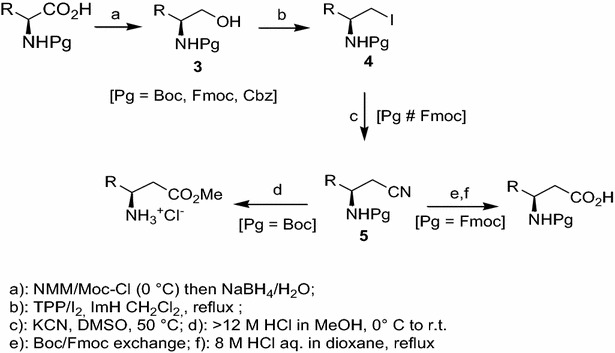
Multistep homologation of α-amino acids to *N*- and/or *C*-protected β^3^-amino acids

A remarkable, unified approach to both syn and anti β^2,3^-amino acids in enantiomerically pure form with natural or unnatural side chain, was reported (Yu et al. 2010). The strategy is based on asymmetric cycloaddition of a nitrone derived from d-gulose to Z or E acrylates, followed by a facile N–O cleaving fragmentation reaction. The availability of convenient synthetic routes to monomers predisposed to form helical structures has accelerated the pace of discoveries involving β-peptide helices. In contrast, a general, scalable synthesis of *syn*-β^2,3^-amino acids from α-amino acids has not been reported so far. Our interests towards non-natural amino acids synthesis began some time ago, when we proposed a procedure for converting α-amino acids into their β^3^-homologues (Caputo et al. 1995). The methodology reported generated valuable synthetic intermediates, such as β-amino alcohols **3**, β-amino iodides **4** and β-amino nitriles **5** (Scheme 1). This homologation procedure has since been enhanced, and the β^3^-amino acids, as well as all homologation intermediates, can be also prepared labeled with isotopic ^2^H, using NaBD_4_ in D_2_O in the reduction step [Caputo and Longobardo 2007].

The homologation intermediate *N*-protected β-amino iodides **4** are extremely useful starting materials for the preparation of new classes of unnatural amino acids (Bolognese et al. 2006; Sureshbabu et al. 2011; Longobardo et al. 2013].

Furthermore, *N*-protected β^3^-amino nitriles **5**, with different amine Pgs, are substrates in biotransformation reactions to give the corresponding amides and/or amino acids catalyzed by nitrilases and nitrile hydratases (Liljeblad and Kanerva 2006; Veitía et al. 2009).

We therefore considered it timely to report a practical synthetic application of intermediates **5** in a novel approach to the simultaneous synthesis of *syn* and *anti*-dialkyl β^2,3^-amino acids (including deuterium labeled compounds) from α-amino acids.

## Methods

Solvents, inorganic salts and organic reagents were purchased from commercial resources and used without further purification unless otherwise noted. All of the compounds for which analytical and spectroscopic data are quoted were homogeneous by TLC. TLC analyses were performed using silica gel plates (E. Merck silica gel 60 F-254) and components were visualized by the following methods: ultraviolet light absorbance, iodine adsorbed on silica gel, and ninhydrin spray. Melting points were measured with a Kofler apparatus and are uncorrected. Column chromatography was carried out on silica gel (E. Merck, 70–230 mesh). THF was dried over Na in presence of benzophenone under an Ar atmosphere. All the compounds were characterized by ^1^H and ^13^C NMR spectroscopy. NMR spectra were recorded using Varian Inova 500 and Bruker DRX-400 spectrometers: chemical shifts are in ppm and *J* coupling constants in Hz. High-resolution ES mass spectra were obtained with a Micromass Q-TOF UltimaTM API. Optical rotations were measured with a Jasco 1010 polarimeter (k = 589 nm). One suitable crystal was mounted at room temperature on a Bruker–Nonius Kappa-CCD diffractometer.

### General procedure for Alkylation of N(Boc)-β^3^-amino nitriles

**Fig. 2:**
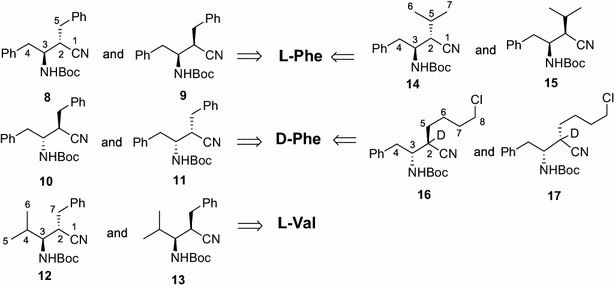
*N*(Boc)-β^2,3^-amino nitriles prepared from l-Phe, d-Phe and l-Val

To a magnetically stirred solution of *N*(Boc)-β^3^-amino nitrile **5** (R^1^ = CH_2_Ph, 1.0 mmol) in anhydrous THF (5 mL), at −78 °C under an argon atmosphere, was added *n*-BuLi (1.6 M in hexane, 2.2 mmol) dropwise over a few minutes. After 15 min, a solution of benzyl bromide (1.5 mmol) in anhydrous THF (1 mL) was added in one portion, and the mixture was allowed to warm to room temperature over 1 h, before diluting with aq. 0.1 M HCl (10 mL) and extracting with EtOAc (3 × 20 mL). The combined organic layers were washed with water until neutral, and dried (Na_2_SO_4_). Evaporation of the solvents under reduced pressure afforded a crude residue consisting of two main products that were readily separated by column chromatography (silica gel, EtOAc-hexane, 1:9). The more mobile product (49 % yield) turned out to be *N*(Boc)-(2*S*,3*S*)-β^2,3^-dibenzylamino propionitrile (**8**, Fig. 2). m.p. = 127–128 °C (from Et_2_O), [α]_D_^25^ = −8.1 (*c* 0.5, CHCl_3_). ^1^H NMR (500 MHz, CDCl_3_) δ: 1.48 (s, 9H, H-Boc), 2.84–3.05 (non resolvable m, 5H, H-2, H-4, H-5), 4.19 (m, 1H, H-3), 4.79 (d, 1H, *J* = 7.8 Hz, H-NBoc), 7.18-7.34 (m, 10H, H-Ar). ^13^C NMR (125 MHz, CDCl_3_) δ: 28.2 (×3), 35.8, 38.8, 40.6, 52.2, 80.2, 119.5, 127.0, 127.2, 128.7 (×4), 128.9 (×4), 136.3, 136.7, 155.3. Anal. Calcd for C_22_H_26_N_2_O_2_ (350.45): C, 75.40; H, 7.48; N, 7.99. Found: C, 75.37; H, 7.50; N, 8.03. HRMS (ESI), exact mass calcd. for [C_22_H_27_N_2_O_2_, 351.2067 (M + H)^+^], found 351.2075. Single crystal of **8** (from Et_2_O solution); data collection at room temperature (MoK_α_ radiation, graphite monocromator, φ and ω scan mode). Crystal data: C_22_H_26_N_2_O_2_, M 350.45, hexagonal, P6_5_, Z 6, a (Å) 11.454(5), b (Å) 11.454(5), c (Å) 27.594(4), V (Å^3^) 3135(2), γ 120.0°, ρ_c_ (g/cm^3)^ 1.114, μ (mm^−1^) 0.071. Semiempirical absorption correction (sadabs) applied. No. of rflns 4817, collected up to θ_max_ 27.52° [No. of independent rflns 2448, No. of rflns with I > 2σ(I) 1068]. The structure was solved by direct methods and anisotropically refined by full matrix least-squares method on F^2^ against all the independent measured reflections. H atoms were geometrically positioned, except for the carbamic H atom that was located in a difference Fourier map, and were refined in a riding model with U_iso_ thermal parameters equal to U_eq_ of the carrier atoms. The final R-factors for 238 refined parameters were R_1_ 0.0717 [on reflections with I > 2σ(I)] and wR_2_ 0.1120 on all the independent reflections. Max and min residual electron densities (eÅ^−3^) +0.117 and −0.120. The absolute configuration (*S*) at C2 was assigned by comparison with the chirality at C3, whose absolute configuration (*S*) is that of the starting l-Phe used for the synthesis of **8**. All crystallographic data were deposited with the Cambridge Crystallographic Data Centre, CCDC No. 632994. The second reaction product (45 % yield) was *N*(Boc)-(2*R*,3*S*)-β^2,3^-dibenzylamino propionitrile (**9**):): m.p. = 132–133 °C (from Et_2_O); [α]_D_^25^ = −43.2 (*c* 0.7, CHCl_3_); ^1^H NMR (500 MHz, CDCl_3_) δ: 1.38 (s, 9H, H-Boc), 2.88 (dd, 1H, *J* = 10.0 and 14.0 Hz, Ha-4), 2.94 (dd, 1H, *J* = 9.1 and 13.4 Hz, Ha-5), 2.97 (dd, 1H, *J* = 4.7 and 13.4 Hz, Hb-5), 3.18 (dd, 1H, *J* = 4.0 and 14.0 Hz, Hb-4), 3.27 (m, 1H, H-2), 4.05 (m, 1H, H-3), 4.60 (d, 1H, *J* = 7.8 Hz, H-NBoc), 7.21–7.39 (m, 10H, H-Ar). ^13^C NMR (125 MHz, CDCl_3_) δ: 28.2 (×3), 35.8, 37.4, 39.6, 51.2, 79.9, 119.9, 127.0, 127.4, 128.9 (×4), 129.2 (×4), 136.3, 136.6, 155.0. The NMR assignments were made using COSY and HSQC experiments. HRMS (ESI), exact mass calcd. for [C_22_H_27_N_2_O_2_, 351.2067 (M + H)^+^], found 351.2059. Under the same conditions, the enantiomer of *N*(Boc)-β^3^-amino nitrile **5** (R^1^ = CH_2_Ph) gave in order: compound **10** (48 %) and compound **11** (43 %). The NMR spectra of **10** and **11** were super imposable to those of compounds **8** and **9**, respectively.

Under the same conditions, from the *N*(Boc)-β^3^-amino nitrile **5** [R_1_ = CH(CH_3_)_2_] and benzyl bromide we obtained:

**12**. (2*S*,3*S*)-3-(*tert*-butoxycarbonylamino)-2-benzyl-4-methylpentanenitrile *anti***12**, 60 %: Amorphous solid; [α]_D_^25^ = +2° (c = 1.1, CHCl_3_); ^1^H-NMR (500 MHz, CDCl_3_) δ: 0.96 and 1.00 (d, 6H, *J* = 6.8 Hz, H-5 and H-6), 1.49 (s, 9H, H-Boc), 1.81 (m, 1H, H-4), 2.93–2.99 (m, 2H, H-7) 2.98-3.04 (m, 1H, H-2), 3.55–3.62 (m, 1H, H-3), 4.70 (d, 1H, *J* = 10.7 Hz, H-NBoc), 7.26–7.38 (m, 5H, H-Ph). ^13^C-NMR (125 MHz, CDCl_3_) δ: 19.1, 19.7, 28.3 (×3), 32.4, 36.5, 38.3, 56.8, 79.9, 119.7, 127.2, 128.7 (×2), 128.9 (×2), 137.1, 155.9. Anal. Calcd for C_18_H_26_N_2_O_2_ (302.41): C, 71.49; H, 8.67; N, 9.26. Found: C, 71.37; H, 8.50; N, 9.23. HRMS (ESI), exact mass calcd. for [C_18_H_27_N_2_O_2_, 303.2067 (M + H)^+^], found 303.2074.

**13**. (2*R*,3*S*)-3-(*tert*-butoxycarbonylamino)-2-benzyl-4-methylpentanenitrile *syn***13**, 30 %: m.p. = 102–103° C (Et_2_O); [α]_D_^25^ = −67° (c = 0.4, CHCl_3_,); ^1^H-NMR (CDCl_3_, 500 MHz) δ: 0.95 and 1.00 (d, 6H, *J* = 6.8 Hz, H-5 and H-6), 1.47 (s, 9H, H-Boc), 2.20–2.30 (m, 1H, H-4), 2.80–2.90 (m, 2H, H-7), 2.88–3.05 (m, 1H, H-2), 3.80–3.90 (m, 1H, H-3), 4.54 (d, *J* = 10.8, 1H, H-NBoc), 7.26–7.36 (m, 5H, H-Ph). ^13^C-NMR (125 MHz, CDCl_3_) δ: 15.5, 20.3, 28.3 (×3), 29.6, 35.4, 38.8, 55.6, 80.1, 119.9, 127.2, 128.8 (×2), 128.9 (×2), 136.9, 155.8. HRMS (ESI), exact mass calcd. for C_18_H_27_N_2_O_2_ 303.2067 [(M + H)^+^], found 303.2052.

Under the same conditions, the alkylation of *N*(Boc)-β^3^-amino nitrile **5** (R_1_ = CH_2_Ph) with isopropyl iodide furnished:

**14**. (2*S*,3*S*)-3-(*tert*-butoxycarbonylamino)-2-isopropyl-4-phenylbutanenitrile *anti***14**, 51 %: m.p. = 91-93 °C (Et_2_O); [α]_D_^25^ = −12,6° (c = 0.2, CHCl_3_); ^1^H-NMR (500 MHz, CDCl_3_) δ: 0.97 and 1.09 (d, 6H, *J* = 6.8 Hz, H-6 and H-7), 1.40 (s, 9H, H-Boc), 1.88–2.00 (m, 1H, H-5), 2.25 (dd, 1H, *J* = 9.0 and 3.2 Hz, H-2), 2.81 (dd, 1H, *J* = 13.7 and 8.8 Hz, H4a), 3.03 (dd, 1H, *J* = 13.7 and 6.8 Hz, H-4b), 4.18–4.27 (m, 1H, H-3), 4.71 (d, 1H, *J* = 10.3 Hz, H-NBoc), 7.20–7.35 (m, 5H, H-Ph). ^13^C NMR (125 MHz, CDCl_3_) δ: 20.5, 20.9, 28.1(×3), 29.6, 41.1, 43.8, 50.4, 79.9, 119.3, 126.9, 128.7 (×2), 129.0 (×2), 136.6, 155.1. Anal. Calcd for C_18_H_26_N_2_O_2_ (302.41): C, 71.49; H, 8.67; N, 9.26. Found: C, 71.37; H, 8.50; N, 9.23. HRMS (ESI), exact mass calcd. for [C_18_H_27_N_2_O_2_, 303.2067 (M + H)^+^], found 303.2049.

**15**. (2*R*,3*S*)-3-(*tert*-butoxycarbonylamino)-2-isopropyl-4-phenylbutanenitrile *syn***15**, 36 %: m.p. = 113–115 °C (Et_2_O); [α]_D_^25^ = −19,7° (c = 0.5, CHCl_3_); ^1^H-NMR (500 MHz, CDCl_3_) δ: 1.10 and 1.16 (d, 6H, *J* = 6.8 Hz, H-6 and H-7), 1.35 (s, 9H, H-Boc), 1.90–2.04 (m, 1H, H-5), 2.65–2.70 (m, 1H, H-4a), 2.77–2.86 (m, 1H, H-4b), 3.13 (dd, *J* = 14.2 and 3.4 Hz, 1H, H-2), 4.12 (m, 1H, H-3), 4.46 (d, 1H, *J* = 8.8 Hz, H-NBoc), 7.20–7.36 (m, 5H, H-Ph). ^13^C NMR (125 MHz, CDCl_3_) *δ*: 19.7, 20.6, 28.1 (×3), 29.6, 37.4, 44.7, 50.3, 79.9, 119.6, 126.8, 128.6 (×2), 129.2 (×2), 136.4, 154.9. HRMS (ESI), exact mass calcd. for [C_18_H_27_N_2_O_2_, 303.2067 (M + H)^+^], found 303.2083.

Under the same conditions, from the di-deuterated enantiomer of *N*(Boc)-β^3^-amino nitrile **5** (R_1_ = CH_2_Ph) (Caputo and Longobardo 2007), alkylation with 4-iodochlorobutane provided:

**16**. (2*R*)-2-[(1*R*)-1-(*tert*-butoxycarbonylamino)-2-phenylethyl]-6-chloro(2-^2^H)hexanenitrile *anti***16**, 56 %: m.p = 75–77 °C; [α]_D_^25^ = −20.5° (c = 0.5, CHCl_3_); ^1^H-NMR (500 MHz, CDCl_3_) δ: 1.41 (s, 9H, H-Boc), 1.58 (m, 4H, H-6 and H-7), 1.72 (m, 2H, H-5), 2.82 (dd, 1H, *J* = 13.7 and 8.8 Hz, H-4a), 3.02 (dd, 1H, *J* = 13.7 and 6.8, H-4b), 3.49 (t, 2H, *J* = 6.3, H-8), 4.09 (m, 1H, H-3), 4.68 (d, 1H, *J* = 10.3 Hz, H-NBoc), 7.20–7.38 (m, 5H, H-Ph). ^13^C-NMR (125 MHz, CDCl_3_) δ: 24.9, 28.5 (×3), 29.1, 32.1 (×2), 40.9, 44.5, 52.5, 80.4, 119.9, 127.3, 129.1 (×2), 129.2 (×2), 136.7, 155.3. Anal. Calcd for C_19_H_26_DClN_2_O_2_ (351.89): C, 64.85; H, 7.45; D, 0.57; Cl, 10.08; N, 7.96. Found: C, 64.67; H, 8.06; N, 7.88. HRMS (ESI), exact mass calcd. for [C_19_H_27_DClN_2_O_2_, 352.1896 (M + H)^+^], found 352.1883.

**17.** (2*S*)-2-[(1*R*)-1-(*tert*-butoxycarbonylamino)-2-phenylethyl]-6-chloro(2-^2^H)hexanenitrile *syn***17**, 28 %, m.p. = 83–85 °C; [α]_D_^25^ = −21.6° (c = 0.65 CHCl_3_); ^1^H-NMR (500 MHz, CDCl_3_) δ: 1.36 (s, 9H, H-Boc), 1.61 (m, 4H, H-6 and H-7), 1.81 (m, 2H, H-5), 2.80–2.90 (m, 1H, H-4a), 3.10 (dd, 1H, *J* = 13.7 and 6.8 Hz, H-4b), 3.54 (t, 2H, *J* = 6.3 Hz, H-8), 3.98–4.02 (m, 1H, H-3), 4.55 (d, 1H, *J* = 10.3 Hz, H-NBoc), 7.22–7.33 (m, 5H, H-Ph). ^13^C-NMR (125 MHz, CDCl_3_) δ: 24.9, 28.4 (×3), 28.5, 28.8, 32.1, 37.3, 44.5, 52.5, 80.4, 120.4, 127.2 (×2), 128.9 (×2), 129.4 136.5, 155.3. HRMS (ESI), exact mass calcd. for [C_19_H_27_DClN_2_O_2_, 352.1896 (M + H)^+^], found 352.1890.

**18**. (2*R*,3*R*)-Methyl 3-Amino-2-benzyl-4-phenylbutanoate hydrochloride, 81 %. Methanolysis of compound **10** in the conditions previously described (Caputo et al. 1995; Caputo and Longobardo 2007] yielded the hydrochloride salt (81 %) as colorless crystals: m.p. 205–207 °C from Et_2_O; [α]_D_^25^ = −21.6 (c 0.2, MeOH). HRMS (ESI), exact mass calcd. for [C_18_H_22_NO_2_, 284.1645 (M + H)^+^], found 284.1658. The ^1^H NMR and ^13^C NMR spectra matched those reported in the literature (Seki et al. 2000) m.p. 208–210 °C; [α]_D_^25^ = −22.5 (c 0.17, MeOH).

## Results and discussion

**Scheme 2:**
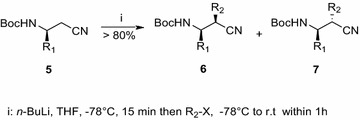
*Anti* (**6**) and *syn* (**7**) *N*(Boc)-α,β-dialkyl β-amino nitriles from *N*(Boc)-β^3^-amino nitriles

LDA and metallated-HMDS which are commonly used to produce α-carbanions from *N*(Boc)-β^3^-amino esters, turned out to be completely ineffective toward *N*(Boc)-protected nitriles **5**. We found that chiral *N*(Boc)-β^3^-amino nitriles are smoothly alkylated at the α-position. The reaction is not at all diastereoselective, hence leads to essentially equimolecular mixtures of *syn* and *anti* di-substituted β-amino nitriles. The diastereomers can be easily separated and individually converted into the corresponding *N*- and/or C-protected β^2,3^-amino acids. The alkylations were carried out at −78 °C in anhydrous THF, using 2.2 equivalents of *n*-BuLi per mole of the starting *N*(Boc)-β^3^-amino nitrile **5**. Under such conditions the resulting lithium dianion is rapidly formed and precipitates as a white solid from THF. These dianions are quite stable below −65 °C, and rapid quenching with alkyl halides afforded almost equimolecular mixtures of both diastereomeric *anti* and *synN*(Boc)-α,β-dialkyl β-amino nitriles (e.g. **6** and **7** in Scheme 2). One example of moderate selectivity (from 60:40 to 95:5 *anti*:*syn*) observed in the alkylation of β^3^-amino nitriles has been reported, but the starting nitriles were fully protected with two bulky benzyl groups at the amino nitrogen atom in that case, therefore cannot be directly compared to our work (Reetz et al. 1994).

Various *N*(Boc)-β^2,3^-amino nitriles with simple alkyl side chains were prepared under the same conditions. The overall yields of the alkylated products were higher than 80 % in all cases. Details of the synthesis of a series *N*(Boc)-β^2,3^-dialkyl amino nitriles derived from l-Phe, d-Phe and l-Val are shown in Fig. 2 and Table 1.

**Table 1:** Enantiopure *N*(Boc)-β^2,3^-dialkyl amino nitriles **8**–**17** (Fig. 2)

Entry	R_1_	R_2_	[μ]^a^	C(2)-	C(3)-	Products	Yields (%)^b^
1	CH_2_Ph	CH_2_Ph	2.95	*S*	*S*	**8**	49
	CH_2_Ph	CH_2_Ph	3.54	*R*	*S*	**9**	45
2	CH_2_Ph	CH_2_Ph	2.95	*R*	*R*	**10**	48
	CH_2_Ph	CH_2_Ph	3.54	*S*	*R*	**11**	43
3	CH(CH_3_)_2_	CH_2_Ph	3.30	*S*	*S*	**12**	60
	CH(CH_3_)_2_	CH_2_Ph	4.40	*R*	*S*	**13**	30
4	CH_2_Ph	CH(CH_3_)_2_	2.62	*S*	*S*	**14**	51
	CH_2_Ph	CH(CH_3_)_2_	4.50	*R*	*S*	**15**	36
5	CH_2_Ph	CH_2_(CH_2_)_3_Cl	2.14	*R*	*R*	**16**	56
	CH_2_Ph	CH_2_(CH_2_)_3_Cl	3.15	*S*	*R*	**17**	28

^a^Dipole moment in Debye, calculated using Chem3D Ultra 8

^b^Yield of isolated products

In particular, *N*(Boc)-β^3^-amino nitriles **5** (R_1_ = CH_2_Ph) was alkylated with benzyl bromide to afford *N*(Boc)-β^2,3^-dibenzylamino nitriles *anti***8** and *syn***9** in 94 % yield. The same alkylation, performed on ent-**5**, (R_1_ = CH_2_Ph from d-Phe) gave 91 % yield of *anti***10 (**ent-**8)** and *syn***11 (**ent-**9**). Alkylation of **5** with isopropyl iodide (R_1_ = CH_2_Ph) gave the corresponding C2-alkylated derivatives **14** and **15** in 87 % yield. Alkylation of **5[**R_1_ = CH(CH_3_)_2_**]** with benzyl bromide provide the *N*(Boc)-β^2,3^-dialkylamino nitriles *anti***12** and *syn***13** in 90 % yield, that are structural isomers of **14** and **15,** which have the same side chains but installed on different C2 and C3 carbons.

Finally, a specially prepared C2-dideuterated *N*(Boc)-β^3^-alkyl amino nitrile **5**, derived from d-Phe, was alkylated with 4-iodochlorobutane yielding diastereomers **16** and **17** (containing one deuterium atom) in 84 % yield. These derivatives contain a side chain useful for further synthetic elaboration, for example in the preparation of β^2,3^-amino acids with a lysine side chain (Langenhan and Gellman 2003).

In all the alkylations studied, single enantiomers were obtained in very good yields after a simple chromatographic separation, as shown in Table 1. The calculation of the dipole moment for compounds **8**–**17**, using the module Chem3D Ultra of ChemDraw software, suggested that for each pair of diastereomers, the *anti*-stereoisomer was the least polar, and we found that they were eluted first from the silica gel chromatographic columns (EtOAc-Hex 1:9).

**Scheme 3:**
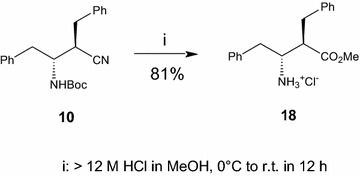
Methanolysis of nitrile **10** to the corresponding methyl ester hydrochloride **18**

**Fig. 3:**
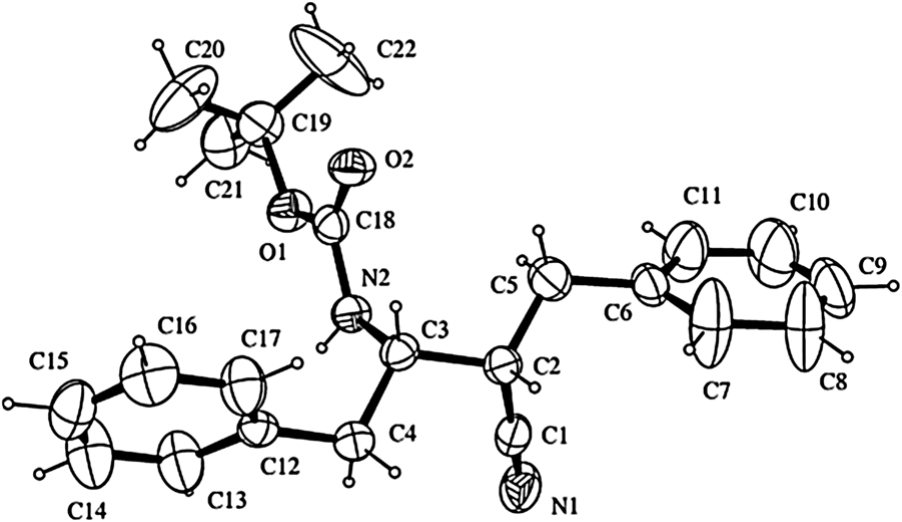
Structure of anti *N*(Boc)-β^2,3^-dibenzylamino nitrile **8** from X-ray structural analysis data

Two of the four *N*(Boc)-β^2,3^-dibenzylamino nitriles, namely **10** and **11** (prepared from d-Phe) could be readily converted into already known amino acids (Seki et al. 2000), which enabled the assignment of the absolute stereochemistry to the chiral centers of compounds **8–11**. Nitrile **10** was converted by methanolysis (14 M HCl in MeOH, 0 °C to room temperature, 12 h) into the corresponding methyl ester hydrochloride **18** in 81 % yield (Scheme 3). The need for a high HCl concentration is necessary for both the transformation of the cyano group and the removal of the amino Pg. The remaining diastereomeric pair, *N*(Boc)-β^2,3^-amino nitriles **8** and **9** (prepared from l-Phe) were enantiomers of **10** and **11**, respectively, and thus could be assigned the opposite configurations at the chiral center. All the stereochemical assignments were eventually confirmed by X-ray analysis of *N*(Boc)-β^2,3^-dibenzylamino nitrile **8** (Fig. 3). The conversions above, along with our existing experience, emphasize the reliability of *N*(Boc)-β^2,3^-amino nitriles as intermediates to afford *N*- and/or *C*-protected β^2,3^-amino acids efficiently, preserving the integrity of the chiral centers.

## Conclusions

The procedure we report here is suitable for the simultaneous preparation of both *syn* and *anti* β^2,3^-amino acids from α-amino acids. The straightforward preparation of the starting chiral *N*(Boc)-protected β^3^-amino nitriles (including deuterium-labeled derivatives) from commercial *N*(Boc)-protected proteinogenic α-amino acids is a noteworthy feature of the whole synthetic scheme. The easy preparation of β^2,3^-amino acids in enantiomeric pure form, with natural or unnatural side chains in position 2 and with the option to introduce other substituents than alkyl groups in this position, starting from low-cost materials, represents a valued synthetic methodology which may find a wide number of applications in the chemistry of amino acids and peptides. In light of the present example, the already mentioned homologation of α-amino acids via β-amino iodides appears to be an effective alternative to the classical Arndt-Eistert homologation procedure, due to the possibility of isolating valuable intermediates, which can be exploited in novel amino acid syntheses.

## Abbreviations

Boc: *tert*-butoxycarbonyl
Cbz: benzyloxycarbonyl
Fmoc: fluorenylmethyloxycarbonyl
ImH: imidazole
HMDS: hexamethyldisilylazane
HMPA: hexamethylphosphoramide
LDA: lithium diisopropylamide
Moc: methyloxycarbonyl
NMM: *N*-methylmorpholine
Pg: protecting group
THF: tetrahydrofuran
TPP: triphenylphosphine
